# Register of food and latex allergy in the Allergy Units of Catalonia

**DOI:** 10.1186/2045-7022-1-S1-O3

**Published:** 2011-08-12

**Authors:** Ramon Lleonart, Maria Basagaña, Vanessa Gázquez, Mar Guilarte, Olga Luengo, Montserrat Molina, Núria Rubira, Laura Valdesoiro

**Affiliations:** 1 Food and Latex Allergy Committee, Catalan Society of Allergy, Barcelona, Spain

## Objective

The number of consultations in the Catalan Allergy Units due to food and latex allergy has increased over the last years. The CIBUS project was created to register the foods that cause allergy with more frequency and the clinical symptoms related to each food and also to latex.

## Material and methods

A specific online data base (CIBUS) was designed. Allergists from 12 different hospitals in Catalonia registered the new diagnoses of food and latex allergy. The cases introduced during the first year have been analyzed.

## Results

278 patients, 232 adults (83.5%) and 46 children (16.5%) have been analyzed. The mean age in adults was 34 years (IQR: 26-44) and in children 5 years (IQR: 3-7). 57.2% were women and 42.8% men. 70% had a personal history of atopic disease.

Milk and egg, 28.3% each, were the more frequently implicated foods in children. In adults; nuts (39.4%), rosaceae fruits (29.9%) and shellfish (11.6%) were the main causes of food allergy. Egg and banana allergy was more frequent in men and anisakis allergy in women.

Urticaria (55.4%) was the most frequent clinical manifestation followed by anaphylaxis (32.4%) and oral allergy syndrome (31.7%). 97.4% of the anaphylactic reactions were presented in adults. Nuts caused 51% of the anaphylactic reactions, rosacea fruits the 35.6% and shellfish the 16.7%. Some foods like shellfish, fruits and nuts caused almost always symptoms while legumes and mustard sensitizations were asymptomatic in most patients. Eleven patients had latex allergy and 4 were sensitized to latex without symptoms. Urticaria was the clinical manifestation in most of the latex allergic patients (72%). Sensitization to airborne allergens was frequent in patients with food allergy 51% of the patients were sensitized to mites, 26.5% to grass, 24.5% to Olea, 18.6% to Platanus, 17.6% to Artemisia and 8.8% to Parietaria pollens.

## Conclusions

The data base is a useful tool to study the characteristics of the food and latex allergy. These data shows that our population presents features similar to the ones described previously. Each food has a different clinical pattern.

